# Novel Botanical Therapeutic NB-02 Effectively Treats Alzheimer’s Neuropathophysiology in an APP/PS1 Mouse Model

**DOI:** 10.1523/ENEURO.0389-20.2021

**Published:** 2021-05-22

**Authors:** Yee Fun Lee, Lavender Lariviere, Alyssa N. Russ, Sang-Zin Choi, Brian J. Bacskai, Ksenia V. Kastanenka

**Affiliations:** ^1^Department of Anatomy and Neurobiology, Boston University School of Medicine, Boston, MA 02118; ^2^Department of Neurology, MassGeneral Institute of Neurodegenerative Diseases, Massachusetts General Hospital and Harvard Medical School, Charlestown, MA 02129; ^3^NeuroBo Pharmaceuticals, Inc, Boston, MA 02116

**Keywords:** Alzheimer’s disease, astrocyte, neuron, therapy

## Abstract

Alzheimer’s disease (AD) is an incurable neurodegenerative disorder and a major cause of dementia. Some of the hallmarks of AD include presence of amyloid plaques in brain parenchyma, calcium dysregulation within individual neurons, and neuroinflammation. A promising therapeutic would reverse or stymie these pathophysiologies in an animal model of AD. We tested the effect of NB-02, previously known as DA-9803, a novel multimodal therapeutic, on amyloid deposition, neuronal calcium regulation and neuroinflammation in 8- to 10-month-old APP/PS1 mice, an animal model of AD. *In vivo* multiphoton microscopy revealed that two-month-long administration of NB-02 halted amyloid plaque deposition and cleared amyloid in the cortex. Postmortem analysis verified NB-02-dependent decrease in plaque deposition in the cortex as well as hippocampus. Furthermore, drug treatment reversed neuronal calcium elevations, thus restoring neuronal function. Finally, NB-02 restored spine density and transformed the morphology of astrocytes as well as microglia to a more phagocytic state, affecting neuroinflammation. NB-02 was effective at reversing AD neuropathophysiology in an animal model. Therefore, in addition to serving as a promising preventative agent, NB-02 holds potential as a treatment for AD in the clinic.

## Significance Statement

No present cure for Alzheimer’s disease (AD) and a great number of clinical trial failures underscores the need for development of therapeutics with multiple mechanisms of action. NB-02 is a multimodal botanical mixture with multiple mechanisms of action. Two-month treatment with NB-02 halts plaque deposition and clears amyloid in cortex as well as hippocampus of APP/PS1 mice. It normalizes neuronal calcium homeostasis, thus restoring neuronal function, and upregulates neuroinflammation. Since NB-02 was effective in slowing pathophysiology in an animal model of amyloidosis, it holds great promise as a therapeutic approach to treat AD.

## Introduction

Alzheimer’s disease (AD) is a progressive neurodegenerative disorder currently without a cure (Holtzman et al., 2011a; Prince et al., 2013). It is characterized by presence of amyloid β deposits in the brain parenchyma (Selkoe, 1994; Hardy and Selkoe, 2002). Circuit dysfunctions underlying cognitive and memory deficits may stem from amyloid β-dependent calcium dyshomeostasis resulting in elevations of baseline calcium within individual neurons (Khachaturian, 1994; Selkoe, 2002). In addition to deposition of amyloid plaques and disruption of calcium homeostasis, AD is characterized by substantial inflammatory response (Gonzalez-Reyes et al., 2017), manifesting in increased expression of inflammatory markers in non-neuronal cells, such as astrocytes and microglia (Araque, 2006; Heneka et al., 2015). Restoration of neuronal calcium to control levels and normalization of neuroinflammatory response would indicate treatment efficacy (Kastanenka et al., 2016; Wang et al., 2018).

NB-02, previously known as DA-9803, is a novel proprietary botanical cocktail containing extracts from *Morus alba* L. and *Poria cocos* (Pagnier et al., 2018). The extract is reproducibly prepared according to a standardized recipe. Currently in preclinical development by NeuroBo Pharmaceuticals, it is a multimodal therapeutic that has been shown to prevent deposition of amyloid plaques in young APP/PS1 (Jankowsky et al., 2004) mice, an animal model of AD (Pagnier et al., 2018). In addition, it was effective at preventing neuronal calcium elevations, thus maintaining circuit integrity in these mice. Finally, it transformed astrocytes and microglia consistent with changes associated with a more phagocytic state. Thus, NB-02 is an ideal candidate to enter the clinic as a preventative therapeutic for AD. However, what is its treatment potential?

To address this question, eight-month-old APP/PS1 mice were treated with daily gavage of 100 mg/kg NB-02 for two months. We tested the therapeutic’s propensity to slow amyloid deposition, restore neuronal calcium homeostasis, and modify neuroinflammation. Interestingly, NB-02 halted amyloid deposition and cleared some amyloid plaques in cortices of drug-treated animals compared with vehicle-treated mice. Furthermore, it restored calcium homeostasis in neurons that started out with elevated baseline calcium levels before treatment onset. Moreover, it increased spine density and modified morphology of astrocytes as well as microglia, thus affecting neuroinflammation. Therefore, NB-02 had a restorative effect on neuronal and non-neuronal cells in an AD mouse model. Thus, it has great potential to restore healthy circuit function and thereby slow cognitive and memory decline in the clinic.

## Material and Methods

### Animals and surgery

Transgenic APPswe/PS1dE9 mice were used (10 males, 11 females; B6:C3-Tg(APPswe, PSEN1dE9)85Dbo/Mmjax background; https://www.alzforum.org/research-models/appswepsen1de9-line-85). These animals overexpress the Swedish mutation in the human *APP* gene and deltaE9 mutation in the *Presenilin* gene (Jankowsky et al., 2004). All the studies complied with the institutional IACUC Committee as well as National Institutes of Health *Guidelines for the Use of Laboratory Animals*.

APP/PS1 mice underwent intracortical virus injections followed by cranial window installations over the right hemisphere (Levasseur et al., 1975) on the same day starting at seven months of age similar to other reports (Kastanenka et al., 2016, 2017; Pagnier et al., 2018; Wang et al., 2018). Following anesthesia induction, animals were secured in a stereotaxic apparatus. After disinfection of skin, incision was made, skull was cleared and burr holes were drilled through the skull. Using a 10-μl Hamilton syringe, 3 μl of AAV8-YellowCameleon 3.6 (YC3.6; U.Penn Vector Core) was injected in the cortex with the following coordinates: anterior-posterior −1.5, medial-lateral −2, dorsal-ventral −0.8 at the rate of 130 nl/min. Thus, excitatory neurons in right somatosensory cortex were targeted. YC3.6 is a genetically encoded calcium indicator (GECI) that allows determination of absolute intracellular calcium concentration based on its ratio of yellow fluorescent protein (YFP)/cyan fluorescent protein (CFP) in individual cells (Nagai et al., 2004). Viral injections were directly followed by craniotomies over the injection sites. Cranial windows with 5 mm in diameter were fixed with a mixture of dental cement and crazy glue. Body temperature of each animal was maintained through the entire surgical procedure and during the recovery from anesthesia. YC3.6 was allowed to express for a month before imaging with multiphoton microscopy. This also allowed the inflammatory response associated with craniotomies to subside. Drug treatment and multiphoton imaging commenced when animals reached eight months of age.

### Therapeutic treatment

APP/PS1 mice were randomly assigned to the vehicle (five males, five females) or the NB-02 (five males, six females) treatment conditions. Vehicle consisted of hydroxypropyl methyl cellulose (HPMC) dissolved in water. NB-02 drug consisted of NB-02 mixed with vehicle solution. Drug or vehicle treatment was initiated subsequent to the baseline imaging session. Treatment was administered to the animals via daily gavage with 300 μl of 100 mg/kg NB-02 or vehicle for the duration of two months. Drug treatment had no significant effect on animal attrition (data not shown). Treatment continued while the animals were imaged at one- and two-month time points after treatment onset. Following the last imaging session, animals received their last gavage treatment, after which mice were euthanized and perfused transcardially with PBS. Their brains were isolated and processed for immunohistochemistry or biochemistry. Ten animals were treated with vehicle, 11 animals were treated with NB-02, totaling 21 mice.

### Imaging using multiphoton microscopy

Before the baseline imaging session, animals received intraperitoneal injections of 4 mg/kg methoxy-XO4, which crosses the blood-brain barrier and binds to amyloid plaques allowing visualization by multiphoton microscopy (Bacskai et al., 2002; Klunk et al., 2002; Garcia-Alloza et al., 2006). Animals were anesthetized with 2% isoflurane and secured in a stereotactic stage. Intravenous administration of Texas Red dextran into the retro-orbital sinus provided a fluorescent angiogram. Multiphoton microscopy allowed imaging of the angiogram, amyloid plaques and YC3.6-expressing neurons using an Olympus Fluoview 1000MPE mounted on an Olympus BX61WI upright microscope. Same fields of view were identified during all three imaging sessions (baseline, one month, two months) using the landmarks present in the angiogram collected during the baseline imaging session. Images were acquired using a 25× water immersion objective (NA = 1.05). A mode-locked titanium:sapphire laser (MaiTai; Spectra-Physics) generated two-photon fluorescence with either 800- or 860-nm excitation. Amyloid plaques were imaged using 800-nm excitation at 1× zoom. Z stacks were acquired at 5-μm step size. YC3.6 was imaged using 860-nm excitation at 2×, and 5× zoom. These z stacks were acquired at 1-μm step size. Laser power was limited below 30 mW to prevent phototoxicity. Animal body temperature was maintained with a heating pad throughout imaging. Methoxy-XO4 was re-administered before each imaging session to identify newly appeared amyloid plaques.

At the end of each imaging session, animals were allowed to recover from anesthesia while their body temperature was maintained with a heating pad. At the end of the last imaging session, mice received their last gavage treatment and their CSF was collected (see below). Then mice were euthanized using CO_2_. Their bodies were perfused with PBS, and brains isolated. Left hemibrains (that had not contained cranial windows) were fixed with 4% paraformaldehyde and cryoprotected with 15% glycerol overnight. Optimal cutting temperature compound (OCT) was used to freeze the hemibrains, which were subsequently cut into 20-μm coronal sections on a cryostat and mounted onto slides for immunohistochemical processing. Right hemibrains (that had contained cranial windows) were flash frozen in liquid nitrogen and subsequently processed for biochemistry.

### CSF collection

Before euthanasia, each animal was anesthetized with 2% isoflurane and secured in a stereotaxic apparatus. Cisterna magna containing CSF was identified. Dura was punctured with a 30-gauge needle. CSF was collected using P20 pipette using dissection microscope. CSF was stored in low protein binding tubes in −80°C before use.

### Data analysis

Analysis was performed using ImageJ software (http://rsbweb.nih.gov/ij/) to determine amyloid plaque numbers, amyloid plaque burden, and resting calcium levels within individual neuronal processes, or neurites. Since the same fields of view were imaged over time, the same amyloid plaques and neuronal processes could be tracked longitudinally to determine amyloid plaque burden and intracellular calcium changes in each individual neuronal process. To determine amyloid plaque numbers and amyloid plaque burden, each z-stack was processed into a maximum intensity projection. Amyloid plaques were counted manually in each projected image to determine the amyloid plaque number. Each projected image was thresholded, segmented, and the percentage area occupied by amyloid was measured to calculate the amyloid burden. The signal from the amyloid present in blood vessels, cerebral amyloid angiopathy, was excluded from analysis.

The images containing neuronal processes expressing YC3.6 were also analyzed using ImageJ. YC3.6 is a FRET probe, where a donor, CFP, and an acceptor, YFP, are connected by a linker (Nagai et al., 2004). Intracellular calcium concentrations were determined from the ratio of YFP to CFP. The higher the calcium concentration, the greater the YFP to CFP ratio. The background for each channel was calculated by the mode of the intensities of the last slice of each volume. That value was subtracted from its channel. A median filter with a radius of 2 was applied to the fluorescence intensities. YFP was divided by CFP, thus creating a ratio image that could be converted to absolute calcium concentration. Neurons were identified and their processes selected manually as regions of interest (ROIs) using the “free hand” tool on ImageJ in the YFP images. These neuronal ROIs were then exported to the ratio images and the YFP/CFP ratios calculated. The relative change in YFP/CFP ratio (Δ*R*/*R_i_*) was calculated by tracking the same neurites throughout all three imaging sessions. YFP/CFP ratios were converted to [Ca^2+^] with standard equations using the *in situ K*_d_ and Hill coefficient for YC3.6 determined earlier (Kastanenka et al., 2017). Matlab was used to create pseudocolored images based on the calcium concentration using the empirical *R*_min_ and *R*_max_. The ratio values were used to determine the hue and saturation (color) and the brightness values were used to assign the value (intensity) in the pseudocolored images.

For the purpose of analyzing dendritic spines, the same YC3.6 multiphoton images at 5× magnification were used. Planes within the z stack were individually selected, dendrites were traced for length using ImageJ. Spines along each dendrite were counted. Spine density was determined as the number of spines divided by corresponding dendrite length. Dendrite segments ranged 8–47 μm in length.

### Immunohistochemistry and image analysis

Twenty-micrometer coronal sections of mouse brains were subjected to antigen retrieval in citrate buffer. The sections were then permeabilized using Triton X-100, blocked with normal goat serum (NGS), and incubated with the following primary antibodies: 82E1 (mouse anti-82E1, 1:500, IBL 10323), glial fibrillary acidic protein (GFAP; mouse monoclonal anti-GFAP, 1:200; Sigma G3893), or Iba-1 (rabbit monoclonal anti-Iba1, 1:200; ab178846) at 4°C overnight. This was followed by incubation with the respective secondary antibodies (1:500) for 1 h at room temperature. The slides were then mounted with Vectashield antifade mounting media (Vector Laboratories) either with or without DAPI.

For amyloid plaque burden analysis, 20-μm sections with methoxy-XO4-labeled plaques and ex vivo 82E1 immunostaining were imaged using an inverted Zeiss microscope with a 10× objective. Images were thresholded and amyloid burden was calculated as a percentage of the cortical or hippocampal area.

Coronal hemibrain sections stained with GFAP or Iba-1 were imaged using an inverted Zeiss microscope at 10× for the purpose of glial cell counts. ROIs were drawn of the whole hippocampus or a randomly selected section of cortex using the ImageJ free hand or “rectangle” tools, respectively. Cells within the ROI were manually counted while blinded to condition to minimize bias. Representative images were taken with 20× objective.

GFAP and Iba-1-stained sections were also imaged using an inverted Olympus confocal microscope with a 40× objective for the purpose of morphology analysis. ImageJ was used to analyze markers of cell morphology such as process length, cell body diameter, soma area, and shape. Process length was measured as distance from the edge of the soma to the end of the process. Cell body diameter was measured using the Feret’s diameter, the maximum caliper of the cell.

### Biochemical analyses

Each flash-frozen hemibrain was homogenized in ice-cold tris-buffered saline (TBS) in the presence of phosphatase inhibitor and centrifuged at 25,500 rpm for 30 min at 4°C. Supernatants were collected as the TBS soluble fraction and stored at −80°C until use in subsequent analyses. A bicinchoninic acid assay (Pierce BCA, Thermo Fisher Scientific) was performed for determination of total protein concentration in each sample.

Total soluble amyloid β 40 and 42 levels were measured in the TBS soluble fraction and in CSF samples using amyloid β 40 and 42 sandwich ELISA kits (Wako) according to manufacturer instructions. Amyloid β 40 and 42 levels were determined using their respective standard calibration curves and protein levels were normalized to wet brain weights.

Synaptic protein levels were measured by Western blotting. Briefly, 35 μg of total protein per sample were prepared in NuPAGE LDS buffer and reducing agent (Invitrogen) then loaded on a 4–12% NuPAGE Bis-Tris Gel (Invitrogen) and run in MES buffer (Invitrogen). Proteins were then transferred to PVDF membranes using a wet blotting system (Mini Blot Module, Invitrogen). Membranes were incubated overnight at 4°C with the primary antibodies: rabbit anti-PSD95 (1:1000, Proteintech, 20 665–1-AP), mouse anti-synaptophysin (clone SY38, 1:2000, Abcam, ab8049), and mouse anti-β-tubulin I (1:5000, Sigma-Aldrich, T7816) as control. The following day, blots were incubated with infrared secondaries IRDye800 or IRDye680 (1:5000, LICOR) for 1 h at room temperature. Blots were then imaged using the Odyssey Infrared Imaging System (LICOR), converted to greyscale, and analyzed using Image Studio Lite Ver5.2. Protein levels were normalized to loading control protein levels.

Proinflammatory cytokine levels (IL-10, TNF-α, IL-6, IL-1β) and chemokine levels (KC/GRO, MCP-1) were measured by electrochemiluminescence immunoassays from Meso Scale Discovery (MSD) according to manufacturer instructions with small modifications as described previously (Bachstetter et al., 2012). Cytokine and chemokine levels were determined by appropriate standard calibration curves using Discovery Workbench 4.0 (MSD) and protein levels were normalized to total protein.

### Statistics

GraphPad 5.0 was used to run statistical analyses. Data were represented as mean ± SEM. Datasets were tested for normality using the D’Agostino and Pearson omnibus normality test, Shapiro–Wilk normality test, or Kolmogorov–Smirnov test. Normally distributed datasets were subjected to parametric tests, such as *t* tests or ANOVAs. Nonparametric datasets were compared using nonparametric tests, such as Mann–Whitney or Kruskal–Wallis tests. Repeated measures datasets were compared using nonparametric Wilcoxon matched pairs signed-rank test; *p* < 0.05 was considered significant.

## Results

### NB-02 is effective at halting amyloid plaque deposition in old APP/PS1 mice

**Figure 1.**
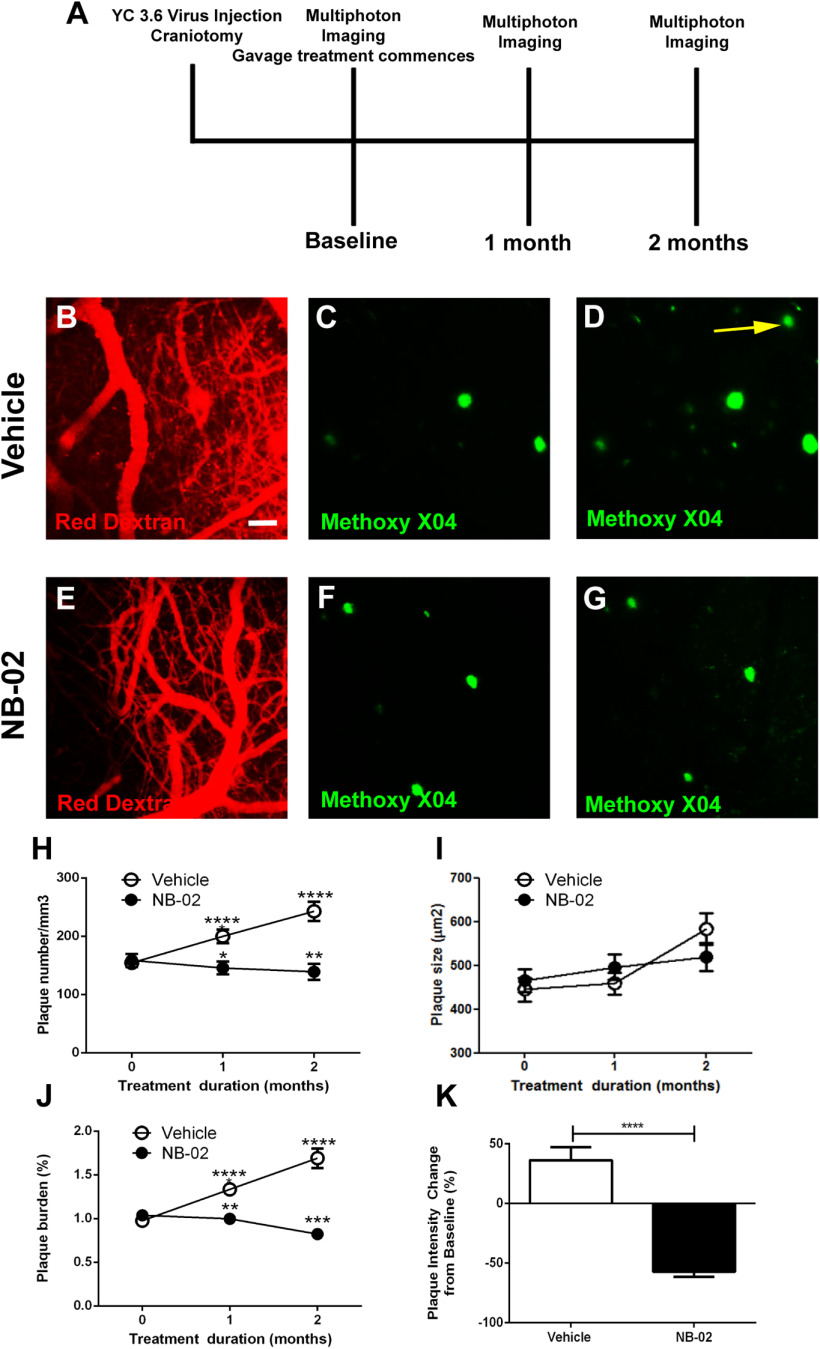
Chronic treatment with NB-02 halts deposition of cortical amyloid plaques *in vivo*. ***A*,** Experimental schematic showing the time-points of viral injections, craniotomies, multiphoton imaging sessions, and gavage treatments with NB-02 or vehicle of APP/PS1 mice. ***B***, ***E***, Multiphoton microscopy images of red dextran angiograms in APP/PS1 mice before treatments with vehicle (***B***) or NB-02 (***E***). ***C***, ***F***, Multiphoton microscopy images of methoxy-X04-positive amyloid plaques in cortices of APP/PS1 mice before treatments. Note ***B***, ***C*** were taken during the same imaging session and constitute the same field of view. Also, ***E***, ***F*** were taken during the same imaging session and constitute the same field of view. ***D***, ***G***, Images of amyloid plaques taken after treatments with vehicle or NB-02. Note ***C***, ***D*** were taken during different imaging sessions and constitute the same field of view. Similarly, ***F***, ***G*** were taken during different imaging sessions and constitute the same field of view. ***H***, Plaque numbers per cubic millimeter of cortex across the two conditions over time. Statistical comparisons are made to baseline (0 months). ***I***, Size of amyloid plaques in the course of treatment across conditions. ***J***, Amyloid plaque burden, which takes into account plaque number and size, over time across conditions ***K***, Methoxy-X04 intensity change at the end of treatment. Scale bar: 100 μm. Mean ± SEM; *p < 0.05, **p < 0.01, ***p < 0.001, ****p < 0.0001; n.s., not significant.

NB-02 is a multimodal botanical cocktail containing extracts from *M. alba* L. and *P. cocos*. To test its propensity for treatment of AD pathology *in vivo*, eight-month-old APP/PS1 mice were subjected to daily gavage treatments with 100 mg/kg NB-02 dose or vehicle cocktail (Fig. 1*A*). Methoxy-X04 (Klunk et al., 2002; Kastanenka et al., 2015) was administered intraperitoneally, crossed blood-brain barrier, and bound to amyloid deposits to allow visualization of amyloid plaques within the same fields of view of cranial windows using high-resolution multiphoton microscopy over the course of treatment (Bacskai and Hyman, 2002; Wang et al., 2014; Kastanenka et al., 2015). A fluorescent angiogram was acquired during baseline imaging session after injection of Texas Red Dextran to allow visualization of the field of view and increase reliability of finding the same fields of view during subsequent imaging sessions (Fig. 1*B*,*E*). Daily gavage treatment was initiated on the same day following the baseline imaging session, starting at eight months of age, and multiphoton imaging was repeated one and two months after treatment onset.

Animals randomly assigned to vehicle and drug treatment conditions started with similar numbers of plaques in somatosensory cortex (154 ± 9 plaques/mm^3^ across 62 z-stacks in 10 mice treated with vehicle, 159 ± 11 plaques/mm^3^ across 63 z-stacks in 11 mice treated with NB-02, mean ± SEM, Mann–Whitney test, n.s.). Vehicle-treated animals exhibited increases in the number of amyloid deposits within the cortical fields of view over time (Fig. 1, compare *D* and *C*, yellow arrow). Amyloid plaque numbers increased to 200 ± 12 plaques/mm^3^ in vehicle-treated animals one month after treatment onset, significantly different from baseline (mean ± SEM, Wilcoxon matched pairs signed-rank test, *W* = −990, *p* < 0.0001; Fig. 1*H*). Plaque deposition again increased to 243 ± 16 plaques/mm^3^ two months after treatment onset, significantly different from baseline (mean ± SEM, Wilcoxon matched pairs signed-rank test, *W* = −741, *p* < 0.0001; Fig. 1*H*). Interestingly, NB-02 halted amyloid depositions and resulted in clearance of amyloid plaques in APP/PS1 mice (Fig. 1, compare *G* and *F*). NB-02-treated animals experienced a decrease in plaque numbers per cortical volume at one month after treatment onset (146 ± 11 plaques/mm^3^ mean ± SEM, Wilcoxon matched pairs signed-rank test, *W* = 294, *p* < 0.05; Fig. 1*H*). By two months after treatment onset, the number of amyloid plaques decreased to 139 ± 14 plaques/mm^3^ (Wilcoxon matched pairs signed-rank test, *W* = 227, *p* < 0.01). At the end of treatment period, average plaque number was significantly lower in NB-02-treated animals compared with those treated with vehicle (243 ± 16 plaques/mm^3^ for vehicle, 139 ± 14 plaques/mm^3^ for NB-02, mean ± SEM, Mann–Whitney test, *U* = 308.5, *p* < 0.0001; Fig. 1*H*).

Amyloid plaque burden analysis was performed, taking into account the number and size of plaques. At baseline, vehicle-treated animals exhibited 0.98 ± 0.05% burden (mean ± SEM), while NB-02-treated mice had 1.04 ± 0.06% burden (mean ± SEM, Mann–Whitney test, n.s.). Amyloid plaque burden increased to 1.33 ± 0.07% in vehicle-treated animals one month after treatment onset, significantly higher compared with baseline (mean ± SEM, Wilcoxon matched pairs signed-rank test, *W* = −1770, *p* < 0.0001; Fig. 1*J*). By two months after treatment onset, amyloid plaque burden increased higher to 1.69 ± 0.11%, significantly different from baseline (mean ± SEM, Wilcoxon matched pairs signed-rank test, *W* = −861 *p* < 0.0001; Fig. 1*J*). Complementary to plaque number findings, amyloid plaque burden decreased to 1.0 ± 0.05% in NB-02-treated mice after one month (mean ± SEM, Wilcoxon matched pairs signed-rank test, *W* = 695, *p* < 0.01; Fig. 1*J*) and then decreased further to 0.82 ± 0.06% after two months of treatment with NB-02 (mean ± SEM, Wilcoxon matched pairs signed-rank test, *W* = 491, *p* < 0.001; Fig. 1*J*). Thus, by the end of treatment, vehicle-treated mice had substantially higher amyloid burden (1.69 ± 0.11%) compared with NB-02-treated mice (0.82 ± 0.06%; mean ± SEM, Mann–Whitney test, *U* = 69, *p* < 0.0001; Fig. 1*J*).

Although the plaque size was comparable in vehicle and drug conditions (Fig. 1*I*), treatment with NB-02 led to decreases in intensity of individual plaques (Mann–Whitney test, *U* = 15, *p* < 0.0001; Fig. 1*K*). Since it has been verified that neither NB-02 nor vehicle interfered with methoxy-X04 binding to individual plaques (Pagnier et al., 2018), decreases in plaque intensity signaled bona fide plaque clearance in NB-02 condition.

**Figure 2.**
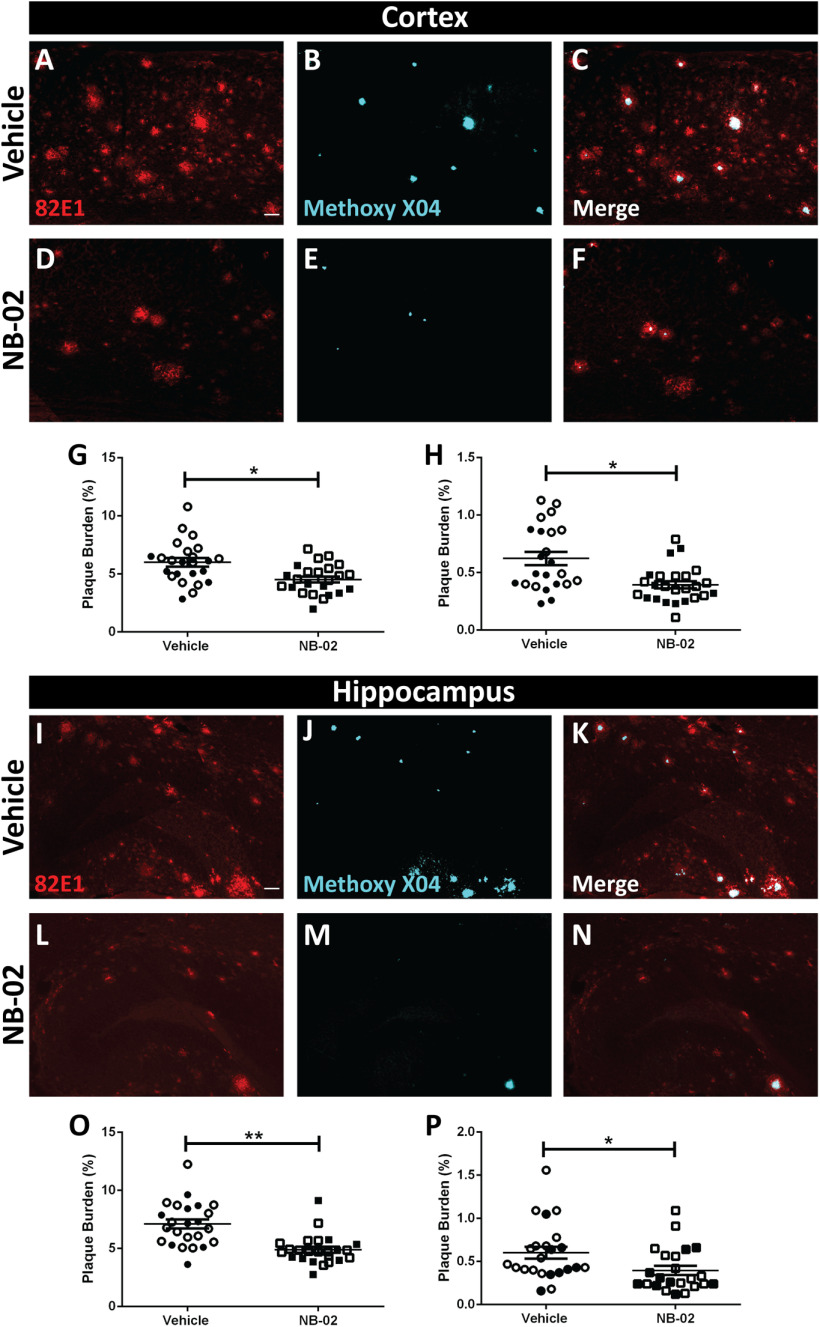
NB-02 treatment results in lower amyloid load compared with vehicle when assessed postmortem. ***A–H***, Cortical images and analysis. ***I–P***, Hippocampal images and analysis. ***A***, ***D***, 82E1 immunoreactivity against amyloid in cortical sections obtained postmortem after treatment with vehicle (***A***) or NB-02 (***D***). ***B***, ***E***, Methoxy-X04-positive amyloid plaques in cortical sections. Images in ***A***, ***B*** were acquired from the same field of view. Similarly, images in ***D***, ***E*** were acquired from the same field of view. ***C***, ***F***, Colocalization of 82E1 and methoxy-X04. ***G*, *H*,** Cortical amyloid plaque burden as assessed by 82E1 immunoreactivity (***G***) and methoxy-X04 signal (***H***). ***I***, ***L***, 82E1 immunoreactivity against amyloid in hippocampal sections obtained postmortem after treatment with vehicle (***I***) or NB-02 (***L***). ***J***, ***M***, Methoxy-X04-positive amyloid plaques in hippocampal sections. Images in ***I***, ***J*** were taken from the same field of view. Similarly, images in ***L***, ***M*** were taken from the same field of view. ***K***, ***N***, Colocalization of 82E1 and methoxy-X04. ***O***, ***P***, Hippocampal amyloid plaque burden as assessed by 82E1 immunoreactivity (***O***) and methoxy-X04 signal (***P***). Each dot represents amyloid burden in a cortical or hippocampal section. Open circles or squares represent females and filled circles or squares represent males. Scale bars: 50 μm. Mean ± SEM; *p < 0.05, **p < 0.01.

As multiphoton microscopy allowed imaging of small cortical regions, we set out to verify the above findings in larger cortical regions using coronal cross-sections of hemibrains processed postmortem following treatment. Brain sections were immunostained with anti-amyloid β antibody 82E1 which recognizes the N-terminus of Aβ but not full-length APP. While methoxy-X04 labeled dense cores, 82E1 decorated the periphery as well as cores of amyloid plaques (Fig. 2*A–F*,*I–N*). Vehicle-treated brains exhibited high amyloid plaque load, evident with 82E1 immunoreactivity and methoxy-X04 labeling in cortex (Fig. 2*A–C*). Intriguingly, NB-02 treatment resulted in decreased cortical amyloid deposition (Fig. 2*D–F*). 82E1 immunoreactivity suggested that amyloid plaque burden was significantly lower as a result of NB-02 treatment compared with vehicle (*t* test, *t*_(14)_ = 2.498, *p* < 0.05, *n* = 24 sections in 8 mice/vehicle; *n* = 24 sections in 8 mice/NB-02; Fig. 2*G*). Similarly, methoxy-X04 labeling indicated lower plaque burden at the end of NB-02 treatment (*t* test, *t*_(14)_ = 2.486, *p* < 0.05, *n* = 23 sections in 8 mice/vehicle; *n* = 25 sections in 8 mice/NB-02; Fig. 2*H*).

Amyloid plaque burden was also calculated in hippocampi of treated mice postmortem (Fig. 2*I–P*). There, 82E1 detected amyloid on the periphery of plaques in addition to their centers, while methoxy-X04 identified dense cores exclusively (Fig. 2*I–N*). Similar to the data in the cortex, NB-02 treatment resulted in decreased amyloid plaque burden as assessed by 82E1 immunoreactivity (*t* test, *t*_(14)_ = 3.252, *p* < 0.01, *n* = 24 sections in 8 mice/vehicle; *n* = 24 sections in 8 mice/NB-02; Fig. 2*O*) or methoxy-X04 labeling (*t* test, *t*_(14)_ = 2.265, *p* < 0.05, *n* = 24 sections in 8 mice/vehicle; *n* = 23 sections in 8 mice/NB-02; Fig. 2*P*). It had previously been verified that neither NB-02 nor vehicle interfered with methoxy-X04 binding to amyloid plaques (Pagnier et al., 2018). Thus, amyloid plaque burden analysis performed postmortem agreed with the *in vivo* data (Fig. 1), where treatment with NB-02 resulted in amyloid plaque clearance in an animal model of amyloidosis.

### NB-02 treatment does not alter soluble amyloid β levels measured with ELISAs in CSF or TBS-soluble brain fractions

**Figure 3.**
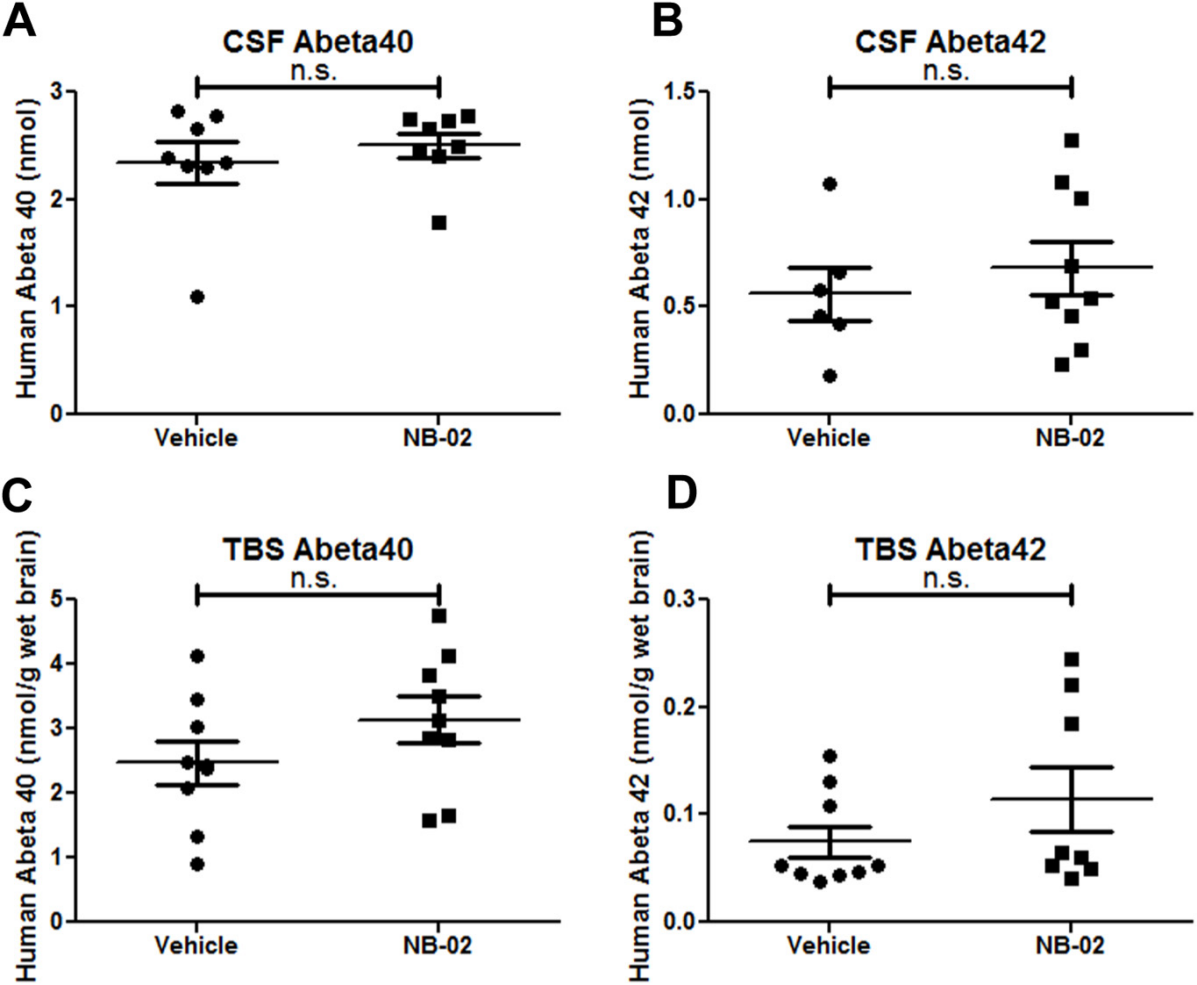
NB-02 does not alter soluble amyloid levels. ***A***, ***B***, Human amyloid β (***A***, 40; ***B***, 42) levels in cerebrospinal fluid (CSF) of APP/PS1 mice treated with vehicle or NB-02. ***C***, ***D***, Human amyloid β (***C***, 40; ***D***, 42) levels in TBS-soluble brain fractions of APP/PS1 mice treated with vehicle or NB-02. Each dot represents amyloid measurement in CSF or brain fraction obtained from individual mice; n.s., not significant.

To determine whether treatment with NB-02 altered soluble amyloid β levels, CSF samples were collected from cisterna magna of APP/PS1 mice at the end of the treatment with the drug or the vehicle. ELISAs specific for amyloid β 40 or 42 were used to measure protein levels. Interestingly, CSF amyloid β 40 levels showed a trend toward an increase in animals treated with NB-02, however failed to reach statistical significance (Fig. 3*A*). Similarly, CSF amyloid β 42 levels measured after NB-02 treatment also tended to increase, however these levels were not statistically different from those after vehicle treatment (Fig. 3*B*).

We also measured TBS-soluble amyloid β 40 and 42 levels in brains of APP/PS1 mice treated with NB-02 or vehicle. Amyloid β 40 levels in TBS soluble fractions trended up in NB-02 condition compared with vehicle (Fig. 3*C*), not reaching significance. Furthermore, TBS-soluble amyloid β 42 levels were not significantly different between the two conditions, yet further supported the trend up in NB-02-treated brains (Fig. 3*D*). Thus, the ELISA results suggest that neither soluble amyloid β 40 nor 42 levels were significantly altered by NB-02 treatment, albeit showing an upward trend.

### NB-02 restores neuronal calcium homeostasis

**Figure 4.**
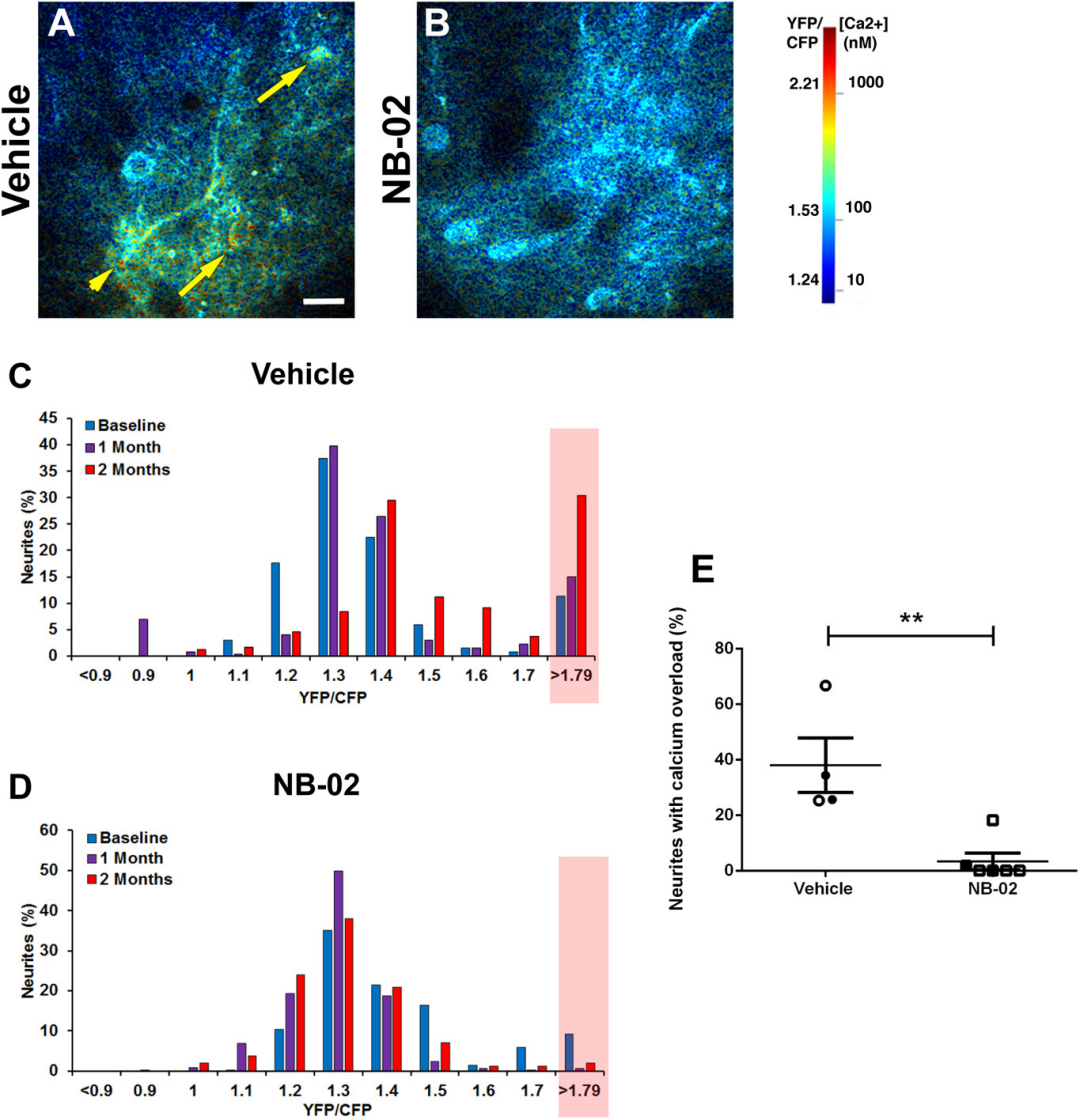
NB-02 restores neuronal calcium overload *in vivo*. ***A***, ***B***, Multiphoton microscopy images of calcium reporter YC3.6 expressed in neurons pseudocolored according to intracellular calcium concentrations in APP/PS1 brains treated with vehicle (***A***) or NB-02 (***B***). Yellow arrows point to neuronal cell bodies exhibiting calcium overload, while the yellow arrowhead points to a neuronal process exhibiting calcium overload. ***C***, ***D***, Histograms showing percentages of neurites binned into categories according to the YFP/CFP ratios over the course of treatment with vehicle (***C***) or NB-02 (***D***). Percentage of neurites with calcium overload, YFP/CFP ratio > 1.79, are shaded in red. ***E***, Bar graph showing percentages of neurites exhibiting calcium overload at the end of treatment. Each dot represents percent calcium overload in an individual mouse. Open circles or squares represent females while filled circles or squares represent males. Scale bar: 50 μm. Mean ± SEM; **p < 0.01.

To determine whether NB-02 treatment has a direct effect on neuronal health, we assessed NB-02’s propensity to restore calcium homeostasis within neuronal processes, neurites. Animal models of amyloidosis contain a small cortical neuronal population that is vulnerable to amyloid β-dependent calcium dysregulation. This results in baseline calcium elevations, calcium overload, within these neurons (Kuchibhotla et al., 2008). Therefore, restoration of neuronal calcium to control levels would serve as a functional indicator of treatment efficacy. Hence, we determined whether gavage treatment with NB-02 would alter neuronal calcium. A genetically encoded calcium indicator, YellowCameleon 3.6 (Nagai et al., 2004) was virally expressed in cortical neurons (Fig. 1*A*) to calculate absolute baseline calcium concentration within each neuron expressing the calcium reporter. Healthy neuronal function is maintained at ∼100 nm intracellular calcium. Amyloid β present in animal models of amyloidosis, such as APP/PS1 mice used here, leads to calcium elevations within a fraction of cortical neurons (Fig. 4*A*, arrows and arrowhead). Calcium overload was defined as YFP/CFP ratio that was 2 standard deviation (SDs) above the mean in healthy wild-type mice and calculated to be >1.79. As anticipated, we saw 11% of neurons exhibiting calcium overload before vehicle treatment onset (Fig. 4*C*, blue bar in red rectangle) and 9% of neurons exhibiting calcium overload before DA-02 treatment commenced (Fig. 4*D*, blue bar in red rectangle), while the majority of neurons exhibited healthy calcium levels, YFP/CFP < 1.79. As vehicle treatment progressed the percentage of neurons exhibiting calcium overload increased, reaching 30% by the end of treatment (Fig. 4*C*). Surprisingly, there was a substantial decrease in the percentage of neurons with calcium overload one month after NB-02 treatment onset (Fig. 4*D*, purple bar in red rectangle). The low percentage of neurons with calcium overload was maintained at two months of treatment (Fig. 4*D*, red bar in red rectangle). At the end of treatment, the percentage of neurites with calcium overload was substantially lower in the NB-02-treated cortices (3.32 ± 3%) compared with those treated with vehicle cocktail (38 ± 10%; Mann–Whitney test, *U* = 0, *p* < 0.01, *N* = up to 272 neurites in 4 mice/vehicle; *N* = up to 368 neurites in 6 mice/NB-02; Fig. 4*E*). Thus, NB-02 treatment was effective at restoring calcium homeostasis and maintaining optimal intracellular calcium levels for healthy neuronal function.

### NB-02 treatment increases spine density

**Figure 5.**
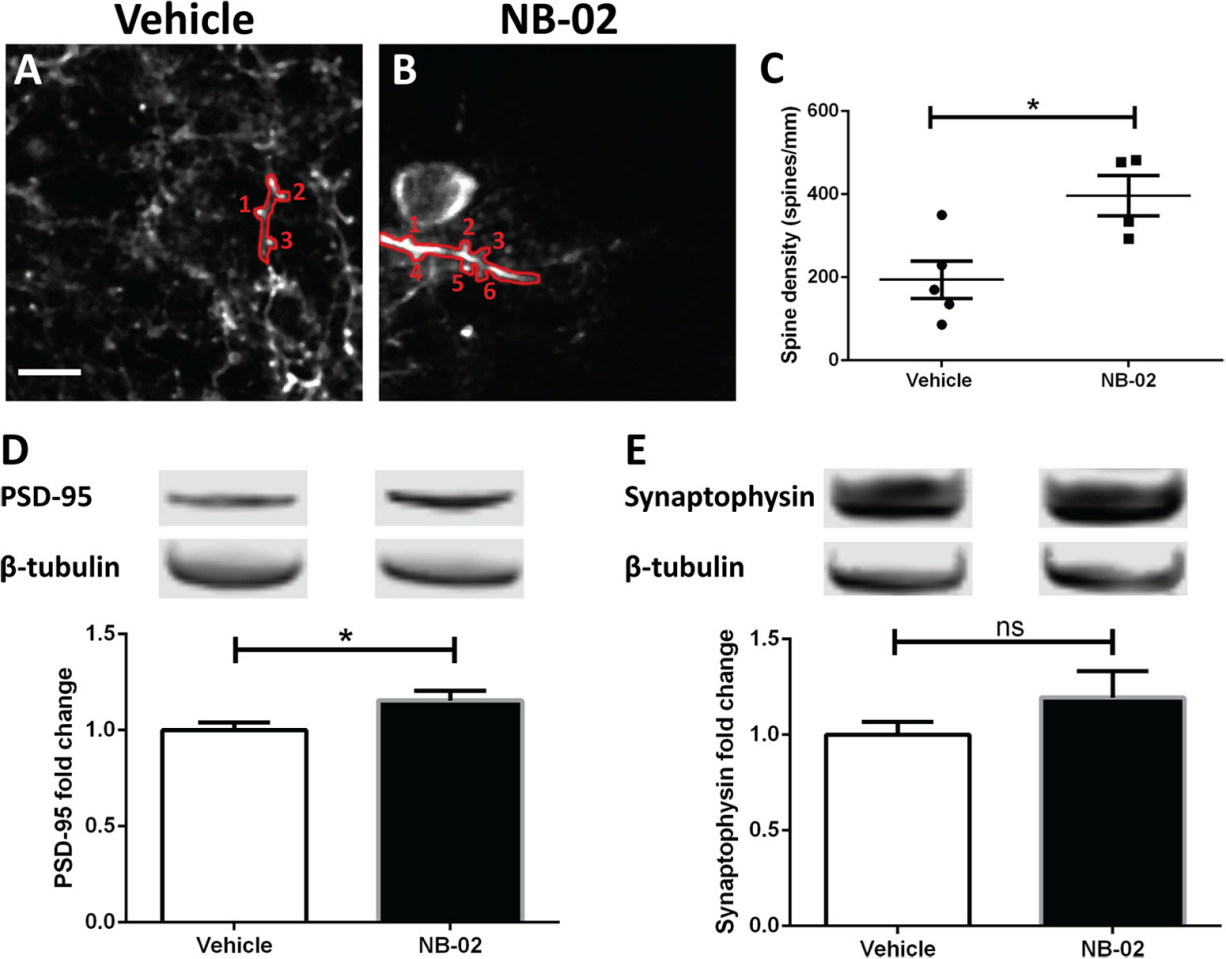
NB-02 increases spine density. ***A*, *B*,** Multiphoton microscopy images of calcium reporter YC3.6 expressed in neurons of APP/PS1 mice treated with vehicle (***A***) or NB-02 (***B***). Individual dendrites are outlined in red and spines are labeled. ***C*,** Spine density assessed posttreatment. Each dot represents an individual mouse. ***D***, ***E***, Bar graphs displaying the comparative levels of synaptic proteins PSD-95 (***D***) and synaptophysin (***E***) assessed by Western blot analyses of vehicle-treated and NB-02-treated brains. Representative bands of PSD-95, synaptophysin, and β-tubulin as control are shown for each condition. Scale bar: 50 μm; *p < 0.05; n.s., not significant.

AD is characterized by loss of synapses, correlated with cognitive decline (Terry et al., 1991; Spires et al., 2005; Spires-Jones et al., 2007). Thus, we assessed synaptic structure with presynaptic and postsynaptic markers and dendritic spine density. Dendritic spines were identified and counted *in vivo* to determine the spine density in vehicle (Fig. 5*A*) and NB-02-treated mice (Fig. 5*B*). Spine density was greater in mice treated with NB-02 compared with those treated with vehicle (*t* test, *t*_(7)_ = 3.025, *p* < 0.05, *n* = 5 mice/vehicle, four mice/NB-02; Fig. 5*C*).

Additionally, synapse structure was assessed using Western blot analyses of the postsynaptic density protein 95 (PSD-95) and the presynaptic protein synaptophysin levels. When compared with the level of PSD-95 in vehicle-treated brains, NB-02 brains had significantly elevated levels of PSD-95 (*t* test, *t*_(15)_ = 2.376, *p* < 0.05, *n* = 9 mice/vehicle and 8 mice/NB-02; Fig. 5*D*). Synaptophysin was analyzed similarly, and though there was a clear upward trend in the NB-02-treated as compared with vehicle-treated brains, no significance was found (*t* test, ns, *n* = 9 mice/vehicle, 8 mice/NB-02; Fig. 5*E*). Thus, NB-02 treatment increases spine density and restores synaptic integrity in APP/PS1 mice.

### NB-02 treatment transforms morphology of glial cells

**Figure 6.**
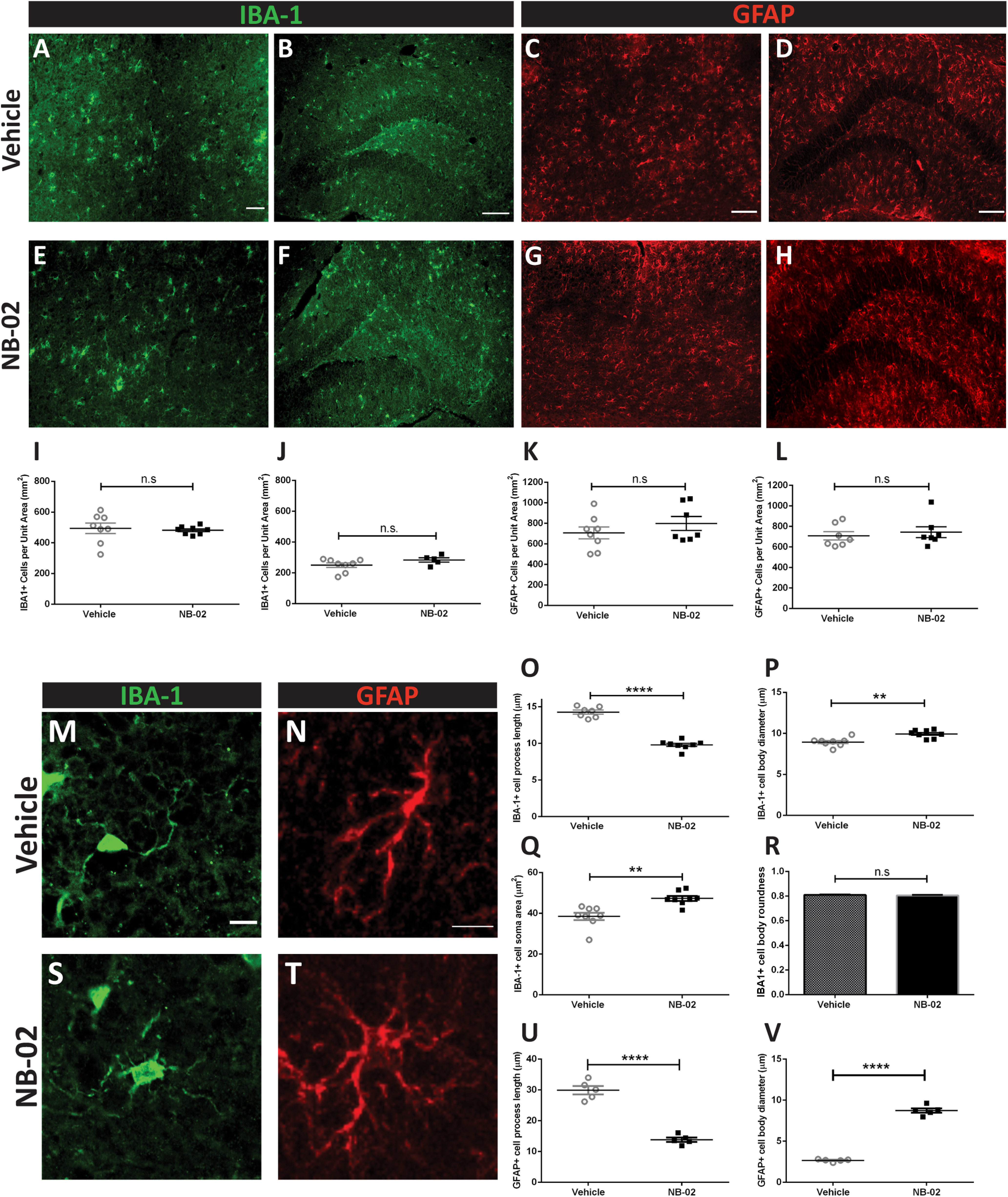
NB-02 transforms morphology of reactive astrocytes and microglia. ***A***, ***E***, Iba-1 immunoreactivity in cortical sections of vehicle-treated (***A***) and NB-02-treated (***E***) APP/PS1 mice. ***B*, *F*,** Iba-1 immunoreactivity in hippocampal sections of vehicle (***B***) and NB-02-treated mice (***F***). ***C***, ***G***, GFAP immunoreactivity in cortical sections of vehicle (***C***) and NB-02-treated mice (***G***). ***D***, ***H***, GFAP immunoreactivity in hippocampal sections of vehicle (***D***) and NB-02-treated mice (***H***). ***I***, Cortical Iba-1-positive cell counts across conditions. ***J***, Hippocampal Iba-1-positive cell counts across conditions. ***K***, GFAP-positive cell counts in cortex across conditions. ***L***, GFAP-positive cell counts in hippocampus. ***M***, ***S***, Higher magnification images of a single microglia in the brain of an APP/PS1 mouse treated with vehicle (***M***) or NB-02 (***S***). ***N***, ***T***, Higher magnification images of a single astrocyte in the brain of an APP/PS1 mouse treated with vehicle (***N***) or NB-02 (***T***). ***O***, Microglia process length across conditions. ***P***, Microglia cell body diameter across conditions. ***Q***, Microglia soma area across conditions. ***R***, Microglia cell roundness across conditions. ***U***, Astrocyte process length across conditions. ***V***, Astrocyte cell body diameter across conditions. Scale bars: 100 μm (***A–D***) and 10 μm (***M***, ***N***). Each dot represents an individual mouse; n.s., not significant; **p < 0.01, ****p < 0.0001.

Neuroinflammation is one of the major hallmarks of AD (Akiyama et al., 2000; Wyss-Coray, 2006; Holtzman et al., 2011b). Thus, the effects of NB-02 on non-neuronal cells such as astrocytes and microglia were analyzed using immunohistochemistry postmortem. The number of cells positive for GFAP (Mucke et al., 1991; Eng and Ghirnikar, 1994) or ionized calcium binding adaptor molecule 1 (Iba-1) were counted in the cortices and hippocampi to determine differences in relative cell numbers at the end of treatment. In addition, morphologic analyses were performed by measuring the length of processes and the size of somas. Upon analyzing cell counts, no significant differences were found in Iba-1-positive cell numbers across conditions for either cortex (*t* test, n.s.; *N* = 8 sections in 8 mice/vehicle, *N* = 8 sections in 8 mice/NB-02; Fig. 6*A*,*E*,*I*) or hippocampus (*t* test, n.s.; *N* = 8 sections in mice/vehicle, *N* = 5 sections in 5 mice/NB-02; Fig. 6*B*,*F*,*J*). Similarly, there were no significant differences in the number of GFAP-positive astrocytes between vehicle and NB-02-treated animals in either cortex (Mann–Whitney test, n.s.; *n* = 8 sections in 8 mice/vehicle, *N* = 7 sections in 7 mice/NB-02; Fig. 6*C*,*G*,*K*) or hippocampus (Mann–Whitney, n.s.; *N* = 7 sections in 7 mice/vehicle, *N* = 7 sections in 7 mice/NB-02; Fig. 6*D*,*H*,*L*).

While no significant differences were found in the numbers of reactive cells, morphologic analyses revealed differences across conditions for both astrocytes and microglia. Astrocytic morphology was altered as a result of NB-02 treatment, as evidenced by shorter processes (*t* test, *t*_(8)_ = 10.5, *p* < 0.0001; *N* = 5 mice/NB-02 with 50 cells and up to 100 processes; Fig. 6*T*,*U*) and larger cell bodies (*t* test, *t*_(8)_ = 21.8, *p* < 0.0001; Fig. 6*T*,*V*). In contrast vehicle-treated astrocytes maintained their long processes and small cell bodies (*N* = 5 mice/vehicle with 50 cells and up to 100 processes; Fig. 6*N*,*U*,*V*). Similarly, morphology of microglia was changed after NB-02 treatment also evidenced by shorter processes (*t* test, *t*_(13)_ = 12.87, *p* < 0.0001; *N* = 8 mice/NB-02 with 414 cells and up to 1080 processes; Fig. 6*S*,*O*) and larger cell bodies, both by diameter (*t* test, *t*_(14)_ = 4, *p* < 0.01; Fig. 6*S*,*P*) and area (Mann–Whitney test, *U* = 3, *p* < 0.01; Fig. 6*S*,*Q*). Microglia in animals treated with vehicle possessed long processes and small cell bodies (*N* = 8 mice/vehicle with 475 cells, up to 1040 processes; Fig. 6*M*,*O–R*). Thus, in addition to affecting neurons, NB-02 treatment had an effect on non-neuronal cells such as astrocytes and microglia.

### NB-02 treatment selectively reduced proinflammatory cytokine levels

**Figure 7.**
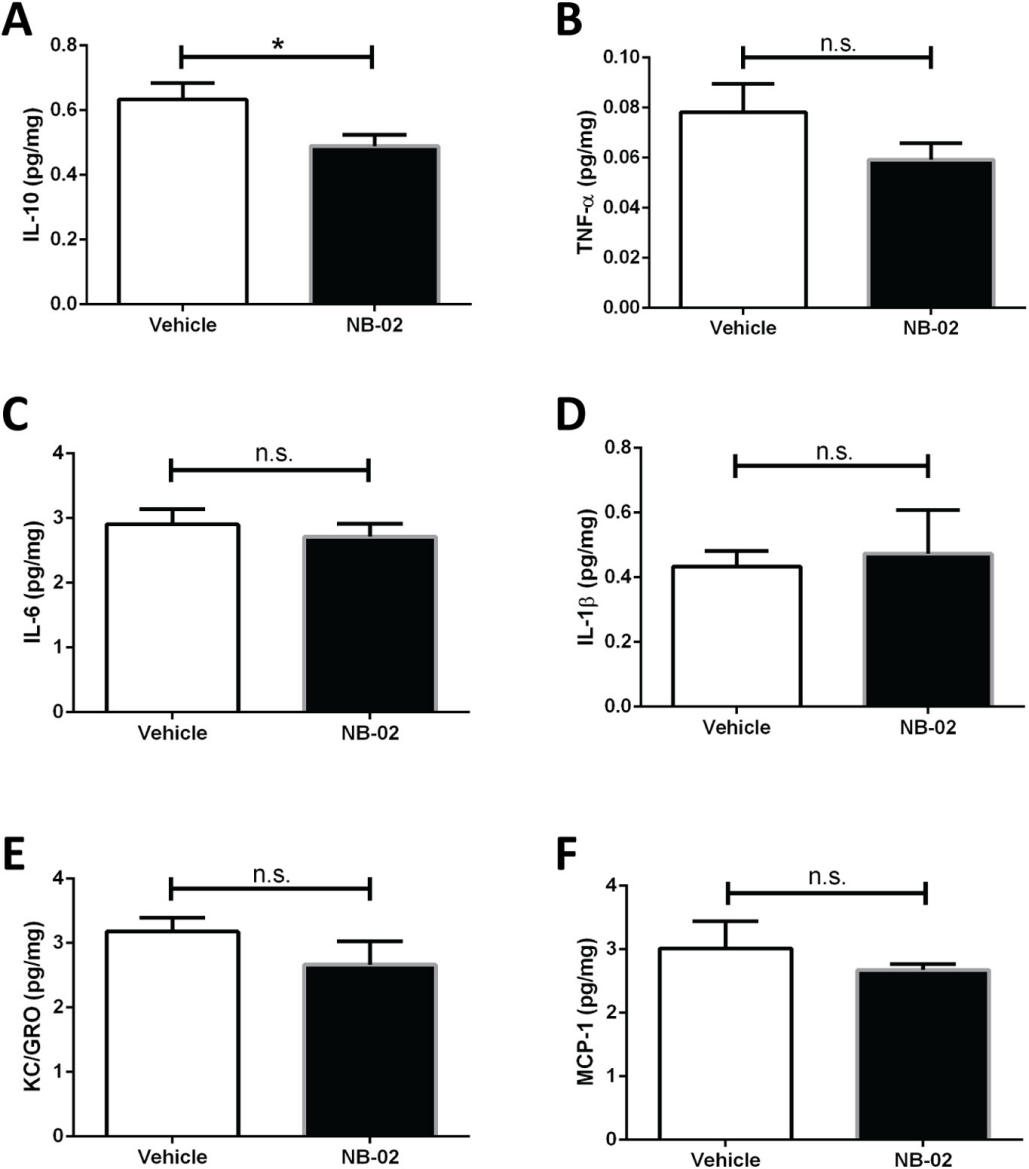
Inflammatory cytokine levels trend down in NB-02-treated brains. ***A–F***, Levels of proinflammatory cytokines IL-10 (***A***), TNF-α (***B***), IL-6 (***C***), IL-1β (***D***), chemokines KC/GRO (***E***), and MCP-1 (***F***) are represented as pg per mg of total protein as assessed by electrochemiluminescent assay; n.s., not significant; *p < 0.05.

To further explore NB-02’s effect on neuroinflammation, the proinflammatory cytokine IL-10, TNFα, IL-6, and IL-1β as well as the chemokine KC/GRO and MCP-1 levels were measured with a highly sensitive electrochemiluminescent immunoassay. IL-10 was significantly reduced in the NB-02-treated compared with vehicle-treated brains (*t* test, *t*_(15)_ = 2.268, *p* < 0.05, *n* = 9 mice/vehicle, 8 mice/NB-02; Fig. 7*A*). Other cytokine and chemokine levels displayed downward trends in the NB-02 condition; however, these failed to reach statistical significance (Fig. 7*B–F*).

## Discussion

NB-02, previously known as DA-9803, is an all-natural botanical drug prepared by mixing natural substances according to a standardized recipe. It has multiple modes of action and has shown great promise as a preventative agent in animal models of AD (Pagnier et al., 2018). Thus, in this study, we assessed its potential as a therapeutic agent.

Starting at eight months of age, APP/PS1 mice were treated with the therapeutic or vehicle via daily gavage for two months. Amyloid plaque deposition was monitored with multiphoton microscopy before, during and after treatment. As early as one month after treatment onset, amyloid deposition was halted and some amyloid cleared in NB-02-treated mice. Animals treated with vehicle continued accumulating amyloid over time. This resulted in a substantially higher number of amyloid plaques in vehicle-treated animals compared with drug-treated mice at the end of treatment. Amyloid burden, which considers the size of plaques as well as their number, was also significantly higher in vehicle-treated mice. Interestingly, amyloid plaques appeared dimmer in NB-02-treated animals; however, this was not because of NB-02 interference of methoxy-X04 binding to the plaques as has been reported previously (Pagnier et al., 2018). Postmortem analysis confirmed elevated amyloid plaque burden in the cortices of vehicle-treated animals as well as their hippocampi. This was important, since multiphoton microscopy allows assessment of amyloid in a small cortical volume. Both mechanisms, decreased plaque deposition and increased plaque clearance, were accountable for the difference in amyloid plaque burden between NB-02-treated and vehicle-treated brains posttreatment. NB-02 might be mediating its effects by decreasing Aβ production, oligomerization, signaling and by increasing clearance. Future studies are needed to elucidate the exact mechanisms of NB-02 action. Additionally, we detected a trend toward increased amyloid β 40 and 42 levels in CSF as well as TBS-soluble fraction of the brains. However, this trend failed to reach significance, possibly because of limited sensitivity of the biochemical procedure. Thus, NB-02 affects extracellular plaques by preventing their deposition and aiding their clearance, possibly by making amyloid more soluble and easier to clear.

Calcium hypothesis of AD posits that disruption of calcium homeostasis contributes to disease progression (Khachaturian, 1994). Disruptions of calcium homeostasis lead to calcium elevations in neurons and astrocytes (Kuchibhotla et al., 2008, 2009). Such elevations have been reported in cell somas as well as cellular compartments such as mitochondria (Calvo-Rodriguez et al., 2020). Aβ-dependent aberrant calcium signaling leads to calcineurin activation and translocation of NFAT into the nucleus (Wu et al., 2012). Calcium elevations have been linked to synaptic loss and cell death (Wu et al., 2010; Calvo-Rodriguez et al., 2020). NB-02 could be targeting any of these signaling pathways. Future studies will elucidate the exact mechanisms of NB-02 action.

APP/PS1 mice contain a population of cortical neurons that is highly vulnerable to amyloid β (Kuchibhotla et al., 2008). Not being able to maintain calcium homeostasis, these neurons exhibit calcium elevations or calcium overload. As such, restoration of baseline neuronal calcium levels would serve as a functional indicator of treatment efficacy (Kastanenka et al., 2016; Pagnier et al., 2018; Wang et al., 2018). Interestingly, NB-02 treatment restored neuronal calcium thus leading to fewer neurons exhibiting calcium overload. The drop in the percentage of neurons with elevated calcium is consistent with decreases in amyloid β reported. Since intracellular calcium supports a myriad of neuronal functions, calcium normalization would signify restoration of these functions, including those supporting cognitive and memory processes of AD patients (Khachaturian, 1994).

Synaptic integrity is essential for healthy circuit function. Synapse loss strongly correlates with performance on neuropsychological tests, measures of cognitive impairment in Alzheimer’s patients, even more so than plaques and tangles (Terry et al., 1991; Masliah et al., 1994). Synapse loss is recapitulated in animal models of amyloidosis (Spires et al., 2005; Spires-Jones et al., 2007), and can be rescued with therapeutic treatment (Wang et al., 2018). NB-02 increased spine density on cortical neurons *in vivo* and resulted in elevations of PSD-95 levels.

Neuroinflammation is an integral process in AD progression (Araque, 2006; Gonzalez-Reyes et al., 2017). Activation of astrocytes and microglia as well as their upregulation of specific receptors has recently been a subject of debate (Kinney et al., 2018; Escartin et al., 2021). Nevertheless, astrocytes and microglia were reported to play an active role in Alzheimer’s progression (Hong et al., 2016; Kastanenka et al., 2020). Thus, we assessed the effect of NB-02 treatment on neuroinflammation. The number of reactive astrocytes and microglia were compared between conditions. However, the number of GFAP-positive astrocytes did not differ significantly in vehicle versus drug-treated mice. Similarly, the number of IBA-1-positive microglia across conditions failed to reach significant difference. Analyses of morphometric changes revealed that, while astrocytes maintained the small cell body and long processes in vehicle-treated animals, NB-02 treatment transformed the morphology of astrocytes, allowing them to assume larger cell bodies and shorter processes. Similarly, microglia morphology also changed, assuming shorter processes and larger cell bodies in the NB-02 treatment condition. Furthermore, NB-02 treatment resulted in decreased IL-10 levels. Thus, NB-02 could be transforming glia morphology and affecting inflammation through direct interactions with glia or indirectly by decreasing Aβ.

In conclusion, NB-02, a botanical therapeutic mixture, is multimodal. It cleared amyloid and resolved neuronal calcium overload, thus restoring neuronal function. Also, it upregulated neuroinflammation, further potentiating amyloid clearance. Furthermore, it restored synaptic integrity. Altogether, this suggests that NB-02 has great potential in the clinic as a treatment for AD.

## Synthesis

Reviewing Editor: Karyn Frick, University of Wisconsin-Milwaukee Decisions are customarily a result of the Reviewing Editor and the peer reviewers coming together and discussing their recommendations until a consensus is reached. When revisions are invited, a fact-based synthesis statement explaining their decision and outlining what is needed to prepare a revision will be listed below. The following reviewer(s) agreed to reveal their identity: Christopher William.

I thank the authors for significantly revising the manuscript to address the reviewer’s comments and critiques. Although one reviewer felt you had adequately addressed their concerns, the other was not available to review your revision. Rather than seeking an additional review and delay a decision, I stepped in to assess the sufficiency of the revision in addressing the reviewer’s concerns. I agree with the first reviewer that the many changes in the revised manuscript, including the additional data, condensed figures, and revised text have greatly improved the manuscript. However, there is one key point that I feel was not adequately addressed, and two critical elements missing from the manuscript that have a major impact on the rigor and transparency of the paper. Each of the items below must be addressed before I can render a final decision.

With regard to the previous review, one reviewer expressed concern that sex of the subjects was not reported, which is a key point because sex is a risk factor for AD and many AD mouse models express sex differences in AD pathology. Although the total number of males and females is now reported in the methods, the number of males and females receiving vehicle vs NB-02 is not. Moreover, with such small N’s (10 male and 11 female of which 10 received vehicle and 11 received NB-02), each treatment group within each sex cannot be more than 5 or 6 (e.g., 5 male vehicle, 5 female vehicle, 6 male NB-02, 5 female NB-02) if the treatments were split evenly by sex. There is no power analysis included in the methods or results, so it is unclear if the studies are sufficiently powered to detect sex differences if they were there. The authors claim in their letter that the results did not differ by sex, but I can find no evidence that any such analyses were ever performed (e.g., sex x treatment or treatment effects within each sex). If they were, and no sex differences were found, then this should be reported. If they were not, then the lack of such analysis should be mentioned and justified.

On a related point, the authors claim to have conducted dozens of statistical analyses of various sorts, but no test statistics are reported, only p values. P values are meaningless without report of the test statistics on which they are based (e.g., the t statistic in a t-test). For example, t-test and ANOVA results should include both the degrees of freedom and the t- or F-statistic, as well as the p value (e.g., t(14) = 3.25, p < 0.05 or F(1,15) = 8.34, p < 0.05), for all significant outcomes. Without this information, readers can not properly evaluate the data on which the author’s claims are based. As such, the authors must add the appropriate test statistics for all statistical tests conducted (e.g., t-test, ANOVA, Mann-Whitney U, Kruskal-Wallis test). This information can be added to the text of the Results section or in tabular form.

A final point is the lack of discussion of the mechanism of action of NB-02. Both this paper and the previous paper by this group on NB-02/DA-9803 (Pagnier et al, 2018) state that NB-02 is a “multimodal botanical", but neither specifies a mechanism of action. The 2018 paper states that “the mechanisms of action of DA-9803 will be investigated in future studies", yet there seems to be no additional information in 2021 about how NB-02 influences AD pathology. It seems clear from this paper and the 2018 publication that it does, but this is an unsatisfying conclusion within having some indication of how it can influence plaques, glia, inflammation, etc. For the research community to take NB-02 seriously as an AD therapeutic, it will be necessary to provide some idea of how it works. Thus, I would like you to add to the discussion some mention of potential neural mechanisms (e.g., binding to X receptors, influencing Abeta uptake and disposal, regulating gene expression) through which NB-02 might influence the outcome measures of this study.

## Author Response

Dear Dr. Karyn Frick:

We thank you and the reviewer for further review of our manuscript titled “Novel botanical therapeutic NB-02 effectively treats Alzheimer’s neuropathophysiology in an APP/PS1 mouse model"(eN-NWR-0389-20). We have addressed the remaining concerns and believe the manuscript is now appropriate for publication. The changes made to the manuscript were tracked. A clean version of the manuscript is also provided. We believe we addressed all the reviewers’ comments. The point-by-point response is as follows.

1. With regard to the previous review, one reviewer expressed concern that sex of the subjects was not reported, which is a key point because sex is a risk factor for AD and many AD mouse models express sex differences in AD pathology. Although the total number of males and females is now reported in the methods, the number of males and females receiving vehicle vs NB-02 is not. Moreover, with such small N’s (10 male and 11 female of which 10 received vehicle and 11 received NB-02), each treatment group within each sex cannot be more than 5 or 6 (e.g., 5 male vehicle, 5 female vehicle, 6 male NB-02, 5 female NB-02) if the treatments were split evenly by sex. There is no power analysis included in the methods or results, so it is unclear if the studies are sufficiently powered to detect sex differences if they were there. The authors claim in their letter that the results did not differ by sex, but I can find no evidence that any such analyses were ever performed (e.g., sex x treatment or treatment effects within each sex). If they were, and no sex differences were found, then this should be reported. If they were not, then the lack of such analysis should be mentioned and justified.

In the first round of revisions, we reported the numbers of males and females in Figure 2G, H, O, P, and Figure 4E as different symbols in the graphs. We have now included the numbers of males and females in the methods section for vehicle (5 males, 5 females) and the NB-02 (5 males, 6 females) as well (line 91-92). Due to the low Ns, we were not able to appropriately assess statistical differences between males and females. For instance, we had two males and two females in the vehicle condition when assessing neuronal calcium overload. Thus, as can be seen, this study is underpowered. Future studies specifically designed to address gender differences are needed to elucidate the effect of NB-02 on gender.

2. On a related point, the authors claim to have conducted dozens of statistical analyses of various sorts, but no test statistics are reported, only p values. P values are meaningless without report of the test statistics on which they are based (e.g., the t statistic in a t-test). For example, t-test and ANOVA results should include both the degrees of freedom and the t- or F-statistic, as well as the p value (e.g., t(14) = 3.25, p < 0.05 or F(1,15) = 8.34, p < 0.05), for all significant outcomes. Without this information, readers can not properly evaluate the data on which the author’s claims are based. As such, the authors must add the appropriate test statistics for all statistical tests conducted (e.g., t-test, ANOVA, Mann-Whitney U, Kruskal-Wallis test). This information can be added to the text of the Results section or in tabular form.

The way statistical analyses were approached was we first verified whether a given dataset was normally distributed. Normally distributed datasets were subjected to parametric tests such as t-test and ANOVA. Datasets that did not follow a Gaussian distribution, were subjected to non-parametric tests such as Mann-Whitney and Kruskal-Wallis tests. We have now reported the statistics and degrees of freedom for the appropriate tests used throughout the results section.

3. A final point is the lack of discussion of the mechanism of action of NB-02. Both this paper and the previous paper by this group on NB-02/DA-9803 (Pagnier et al, 2018) state that NB-02 is a “multimodal botanical", but neither specifies a mechanism of action. The 2018 paper states that “the mechanisms of action of DA-9803 will be investigated in future studies", yet there seems to be no additional information in 2021 about how NB-02 influences AD pathology. It seems clear from this paper and the 2018 publication that it does, but this is an unsatisfying conclusion within having some indication of how it can influence plaques, glia, inflammation, etc. For the research community to take NB-02 seriously as an AD therapeutic, it will be necessary to provide some idea of how it works. Thus, some mention of potential neural mechanisms (e.g., binding to X receptors, influencing Abeta uptake and disposal, regulating gene expression) through which NB-02 might influence the outcome measures of this study should be added to the discussion.

NB-02 is a proprietary botanical drug that is currently in preclinical development, thus my team and I are limited in the amount of information we are able to reveal regarding the drug. However, to address potential mechanisms of action we included mention of how this drug could be affecting amyloid, calcium overload and neuroinflammation. The bottom line is future studies are needed to figure out exactly how the drug works.

Sincerely,

Ksenia Kastanenka
